# *Toxoplasma gondii* – Prevalence and Risk Factors in HIV-infected Patients from Songklanagarind Hospital, Southern Thailand

**DOI:** 10.3389/fmicb.2015.01304

**Published:** 2015-11-25

**Authors:** Waenurama Chemoh, Nongyao Sawangjaroen, Pisut Siripaitoon, Hemah Andiappan, Thanaporn Hortiwakul, Natthawan Sermwittayawong, Bunsri Charoenmak, Veeranoot Nissapatorn

**Affiliations:** ^1^Department of Microbiology, Faculty of Science, Prince of Songkla UniversityHat Yai, Thailand; ^2^Department of Internal Medicine, Faculty of Medicine, Prince of Songkla UniversityHat Yai, Thailand; ^3^Department of Parasitology, Faculty of Medicine, University of MalayaKuala Lumpur, Malaysia

**Keywords:** HIV, IgG avidity, seroprevalence, risk factors, toxoplasmosis

## Abstract

Toxoplasmosis is one of the most common opportunistic parasitic diseases in patients living with HIV/AIDS. This study aimed to determine the seroprevalence of *Toxoplasma* infection in HIV-infected patients and to identify associated risk factors in *Toxoplasma* seropositive patients. This study was conducted at a regional public hospital in Hat Yai, southern Thailand during October 2009 to June 2010. Blood samples were collected from 300 HIV-infected patients. Each subject also answered a socio-demographic and risk factors associated with *Toxoplasma* infection. The prevalence of anti-*Toxoplasma* IgG antibodies in HIV-infected patients was 109 (36.3%), of which 83 (76.2%) had past infection and 26 (23.9%) had recently acquired *Toxoplasma* infection as indicated by their IgG avidity. Multivariate analysis using logistic regression showed that gender difference (adjusted OR = 1.69, 95% CI = 1.05–2.72) was the only factor associated with *Toxoplasma* infection. From the results obtained, these HIV-infected patients could be at high risk of developing clinical evidence of severe toxoplasmosis. Therefore, it is necessary to introduce primary behavioral practices to prevent *Toxoplasma* infection among HIV-infected patients.

## Introduction

Toxoplasmosis is a clinical and/or pathological evidence of a disease caused by *Toxoplasma gondii*, an obligate intracellular protozoan parasite. *Toxoplasma* infection affects about one-third of the world population but the majority of infected individuals are asymptomatic (Montoya and Liesenfeld, 2004). In people who are living with HIV/AIDS, there is an increased risk of reactivation of latent *Toxoplasma* infection in several organs, particularly in the brain leading toxoplasmic encephalitis (TE) that further complicates the course of AIDS (Sukthana, 2006). Globally, the number of patients who died from AIDS has been declining over the years due to the introduction of highly active antiretroviral therapy (HAART). Toxoplasmosis, one of the HIV co-infections, has, however, contributed to the burden of medical care costs (Suwanagool et al., 1997) and to those patients who are repeatedly admitted to government hospitals (Solomon et al., 2006). Till date, approximately 10% of people living with HIV/AIDS are from the Southern part of Thailand (Ministry of Public Health [MOPH], 2011). To the best of our knowledge, there is no epidemiological surveillance reported on the opportunistic infections in HIV-infected patients including toxoplasmosis in this region. Furthermore, IgG avidity testing, a qualitative method, is the first ever serodiagnostic method to be introduced in differentiating chronic from recently acquired *Toxoplasma* infection in these patients. This method definitely helps in better understanding the status of *Toxoplasma* infection and its proper management in HIV patients. It is, therefore, relevant to conduct an epidemiological study of toxoplasmosis by determining the seroprevalence, the association with risk factors and the measurement of IgG avidity from *Toxoplasma* seropositive patients.

## Materials and Methods

### Study Population

This cross-sectional study was carried out at Songklanagarind hospital, Hat Yai, Songkhla province, Thailand with the approval from the ethical committee of the Faculty of Medicine, Prince of Songkla University, Thailand (EC 53-080-14-1-2). The study included 300 HIV-infected patients, who attended the outpatient clinic and/or admitted in the ward at the Department of Internal Medicine during October 2009 to June 2010 and their informed consents were obtained prior to this study. The study subjects were randomly selected from those HIV-infected patients in any age group who had their anti-HIV antibody status examined by screening using the ELISA test and confirmed by the western blot technique. The information on related socio-demographic such as age, sex, and occupation as well as risk factors associated with *Toxoplasma* infection, such as close contact with cats, consumption of uncooked meats, a history of receiving blood transfusion, and some clinical backgrounds such as receiving primary chemoprophylaxis and/or receiving HAART. A CD4 cell count was obtained from their hospital recorded.

### Serum Samples

Approximately 5 mL of venous blood sample was drawn from the participating HIV patients by a venipuncture into a sterile test tube. The sera were obtained after separation by centrifugation at 2500 × *g* for 5 min, and subsequently kept at -20°C until use.

### Detection of anti-*Toxoplasma* IgG antibodies

The serostatus of *Toxoplasma* infection was screened using a standard ELISA commercial kit (IgG-NovaLisa^TM^, Dietzenbach, Germany) in accordance with the manufacturer’s instructions.

### Measurement of IgG avidity

A positive sample for anti-*Toxoplasma* IgG antibody was also tested for its avidity using a standard ELISA commercial kit (IgG-NovaLisa^TM^, Dietzenbach, Germany); high avidity (>40%) indicated a past infection while a low avidity (<40%) indicated a recently acquired infection.

### Statistical Analysis

Data obtained from both the questionnaire and laboratory tests were entered and analyzed using the statistical software SPSS version 10 (SPSS Inc., Chicago, IL, USA). The data with quantitative variables were expressed as a mean (±SD) and range, whereas, qualitative variables were estimated and presented as frequencies and percentages. The Chi-square (χ^2^) test or Fisher exact probability test was chosen to determine the association between possible risk factors and disease transmission. Multivariate analysis adjusted by multiple logistic regressions was used to determine significant differences between demographics or confounding risk factors associated with *Toxoplasma* infection among study subjects. The *p*-value of ≤0.05 was regarded as statistically significant.

## Results

### Demographic Profile of Study Subjects

**Table 1** shows the age range of these HIV-infected patients was 21–78 with a mean of 40.4 ± 8.4 years. Majority of these patients were in the age group of 20–39 years (150, 50%), in addition, they were predominantly married (196, 65.3%), and were laborers (174, 58%). About half of these patients had completed secondary school level of education (153, 51%).

**Table 1:** Univariate analysis of plausible demographic characteristics, clinical profiles, and other possible risk factors associated with *Toxoplasma* seropositive HIV-infected patients.

Demographic characteristics		Number (%)	Number IgG positive (%)	*p*-Value
Age group (years)	20–39 40–59 ≥60	150 (50) 143 (47.7) 7 (2.3)	46 (30.7) 60 (42) 3 (42.9)	0.125^a^
Sex	Male Female	157 (52.3) 143 (47.7)	66 (42) 43 (30.1)	0.031^a^
Marital status	Single Married	104 (34.7) 196 (65.3)	42 (40.4) 67 (34.2)	0.288^a^
Education	Primary Secondary Tertiary	75 (25) 153 (51) 72 (24)	27 (36) 54 (35.3) 28 (38.9)	0.870^a^
Occupation	Laborer Non-laborer Other^b^	174 (58) 59 (19.7) 67 (22.3)	62 (35.6) 24 (40.7) 23 (34.3)	0.728^a^
Present address	Songkhla Outside	149 (49.7) 151 (50.3)	49 (46.2%) 57 (53.8)	0.378^a^
CD4 (cells/cumm)	<200 200–499 ≥500	52 (17.3) 134 (44.7) 114 (38)	22 (42.3) 54 (40.3) 33 (29)	0.111^a^
History of receiving chemoprophylaxis^c^	Yes No	63 (21) 237 (79)	24 (38.1) 85 (35.9)	0.744^a^
History of receiving highly active antiretroviral therapy (HAART)	Yes No	285 (95) 15 (5)	100 (35.1) 9 (60)	0.051^a^
History of toxoplasmic encephalitis (TE)	Yes No	10 (3.3) 290 (96.7)	7 (70) 102 (35.2)	0.024^d^
History of contact with cats	Yes No	191 (63.7) 109 (36.3)	65 (34) 44 (40.4)	0.272^a^
History of eating uncooked meat	Yes No	58 (19.3) 242 (80.7)	20 (34.5) 89 (36.8)	0.744^a^
History of blood transfusion	Yes No	4 (1.3) 296 (98.7)	3 (75) 106 (35.8)	0.106^d^

*^a^*p*-value was evaluated by χ^*2*^ test.*

*^b^Other includes retiree, unemployed, housewives and students.*

*^c^Co-trimoxazole*

*^d^*p*-value was analyzed by Fisher exact probability test.*

### Seroprevalence of Toxoplasmosis in HIV/AIDS Patients and in Association with Demographic Characteristics

The seroprevalence of *Toxoplasma* infection in these patients was 109 (36.3%). Measurement of IgG avidity among *Toxoplasma* seropositive patients showed 83 (76.2%) had high avidity indicates past infection while 26 (23.9%) patients had low avidity indicates recently acquired *Toxoplasma* infection.

Using univariate analysis, this study identified that gender and a history of having cerebral toxoplasmosis were statistically significant factors associated with *Toxoplasma* seropositivity (*p* < 0.05). The data further showed that majority of male patients were significantly found in the age group of 40–59 years, and they make their living through their labor works. It is also shown that these male patients had not received high education, stayed outside the main city and eating of uncooked meat; however, there was no significant association (**Table 2**). Further analysis using multivariate logistic regression showed that gender (male) plays a significant role in *Toxoplasma* seropositivity with adjusted odds ratio of 1.69 (95% CI = 1.05–2.72; **Table 3**).

**Table 2:** Comparison of plausible demographic characteristics, clinical profiles, and other possible risk factors between male and female HIV-infected patients.

Demographic characteristics		Number (%)		*p*-Value
		Male	Female	
Age group (years)	20–39 40–59 ≥60	60 (38.2) 93 (59.2) 4 (2.6)	90 (62.9) 50 (35) 3 (2.1)	0.000^a^
Marital status	Single Married	47 (29.9) 110 (70.1)	57 (39.9) 86 (60.1)	0.092^a^
Education	Primary Secondary Tertiary	33 (21) 84 (53.5) 40 (25.5)	42 (29.4) 69 (48.2) 32 (22.4)	0.248^a^
Occupation	Laborer Non-laborer Other^b^	103 (65.6) 40 (25.5) 14 (8.9)	71 (49.6) 19 (13.3) 53 (37.1)	0.000^a^
Present address	Songkhla Outside	76 (51) 81 (53.6)	73 (49) 70 (46.4)	0.648^a^
History of receiving chemoprophylaxis	Yes No	40 (25.5) 117 (74.5)	23 (16.1) 120 (83.9)	0.064^a^
Seroprevalence of *Toxoplasma* infection	Positive Negative	66 (42) 91 (58)	40 (28) 103 (72)	0.011^a^
History of contact with cats	Yes No	102 (65) 55 (35)	89 (62.2) 54 (37.8)	0.711^a^
History of eating uncooked meat	Yes No	37 (23.6) 120 (76.4)	21 (14.7) 122 (85.3)	0.072^a^
History of blood transfusion	Yes No	1 (0.6) 156 (99.4)	3 (2.1) 140 (97.9)	0.262^c^

*^a^*p*-Value was evaluated by χ^*2*^ test.*

*^b^Other includes retiree, unemployed, housewives and students.*

*^c^*p*-Value was analyzed by Fisher exact probability test.*

**Table 3:** Multivariate logistic regression analysis of risk factors associated with *Toxoplasma* seropositivity.

Demographic characteristics^a^	Adjusted OR (95% CI)^b^	*p*-Value
Gender	1.69 (1.05–2.72)	0.042
History of receiving HAART	0.36 (0.13–1.04)	0.095
History of TE	4.30 (1.09–16.99)	0.075

*^a^Only risk factors with a *p* ≤ 0.10 on univariate analysis were included in multivariate logistic regression analysis.*

*^b^OR, odds ratio; CI, confidence interval.*

### The Prevalence of TE in Patients with AIDS

The past clinical history of these patients showed that 10/300 (3.3%) of HIV/AIDS patients, aged between 28 and 52 years, 5 males, and 5 females, were diagnosed with TE prior to this study. Our study confirmed statistically significant (*p* = 0.024) sero-evidence of anti-*Toxoplasma* (IgG) antibodies in seven cases (data were not shown).

### Other Co-opportunistic Infections in HIV/AIDS Patients

During the time of this study, there was no new diagnosis of TE reported from our patients. However, patients with *Toxoplasma* seropositivity and other concurrent opportunistic diseases were found as follow: 24/72 patients with tuberculosis (TB) had *Toxoplasma* seropositivity and 18 of these TB patients subsequently developed immune reconstitution inflammatory syndrome (IRIS-TB). *Toxoplasma* seropositivity was also found in: 15 patients with herpes virus infections (herpes simplex (HS) and herpes zoster (HZ) viruses), 2 patients with cytomegalovirus (CMV) infections, 2 patients with salmonellosis, 3 patients with histoplasmosis, 5 patients with cryptococcal meningitis, 6 patients with penicillosis, and 4 patients with non-tuberculous meningitis (NTM). Overall, there was no fatal case reported at the end of this study.

## Discussion

Approximately half of HIV-infected patients are co-infected with *T. gondii* (Shimelis et al., 2009; Daryani et al., 2011). In Thailand, previous studies of the prevalence of *Toxoplasma* infection among HIV-infected patients found the prevalence ranging from 22.4 to 53.7% (Wongkamchai et al., 1995; Chintana et al., 1998; Sukthana et al., 2000; Nissapatorn et al., 2001; Wanachiwanawin et al., 2001). In our study, 36.3% of subjects were infected with *Toxoplasma*. This is higher than a study among immunocompetent pregnant women (28.3%) in a study conducted in the same location at the same time (Nissapatorn et al., 2011). Differences in the sensitivity of the ELISA test kit may account for the differences in the prevalence of *Toxoplasma* infection (Chemoh et al., 2013).

IgG avidity testing was developed to avoid the need of conducting confirmatory tests with a second serum sample to determine, if there is a recently acquired infection (Pour Abolghasem et al., 2011). A positive *Toxoplasma* IgG test with a low avidity suggests a recently acquired infection (Liesenfeld et al., 2001; Reis et al., 2006). Based on IgG avidity testing, 8.7% of our subjects had a newly acquired *Toxoplasma* infection and these patients were closely monitored. However, none developed clinical toxoplasmosis during the study.

Using multivariate analysis, male gender was found to be the only significant risk factor for *Toxoplasma* infection. This is consistent with other studies (Nissapatorn et al., 2007; Akanmu et al., 2010). Males were found to be more susceptible to acquire several infections due to their sex steroid hormones that decrease immune responses and influence disease resistance genes and their behaviors (Klein, 2000). These may be the reasons, why the seropositivity of male HIV/AIDS patients to *Toxoplasma* infection was significantly higher than the female HIV/AIDS patients (Roberts et al., 2001). Previously identified risk factors, such as close contact with cats, consumption of uncooked meats and history of receiving a blood transfusion were not significantly associated with *Toxoplasma* infection in our study.

Reactivation of latent *Toxoplasma* infection is common in immunocompromised hosts (Dahnert, 2003) making HIV patients at higher risk for clinical toxoplasmosis. TE is the most common neurological condition (42%) in HIV-infected patients (Ramirez-Crescencio and Velasquez-Perez, 2011). Ten subjects (3.3%) in our study had a previous history of TE as diagnosed by a combination of clinical TE: headaches, seizure, focal neurological deficits, histology and response of therapy, of these 70% (7) were seropositive for *Toxoplasma* infection. This finding is similar to another study who found some of the patients with confirmed TE were not positive for *Toxoplasma* antibodies (Skiest et al., 2000).

## Conclusion

Latent toxoplasmosis is still prevalent in our study population. Gender was the only significant risk factor for *Toxoplasma* infection in our study. Although the number of HIV infected patients in Thailand has decreased nationwide (Ministry of Public Health [MOPH], 2011), we recommend newly infected HIV patients be warned about this opportunistic parasitic disease during the counseling period since both newly acquired and chronic *Toxoplasma* infections in patients with AIDS are occasionally developed clinical toxoplasmosis (Centers for Disease Control and Prevention [CDC], 2009).

## Author Contributions

VN, NS, and PS designed the study. WC, HA, TH, NS, and BC carried out the experiment. WC, NS, and VN helped in manuscript writing and editing. VN, NS, and PS provided opinions and suggestions about this manuscript. All authors read and approved the final version of the manuscript.

## Conflict of Interest Statement

The authors declare that the research was conducted in the absence of any commercial or financial relationships that could be construed as a potential conflict of interest.
